# First clinical study data from therapeutic use of a novel continuous glucose monitoring system in the ICU

**DOI:** 10.1186/cc13633

**Published:** 2014-03-17

**Authors:** N Scawn, N Coulson, I Kemp, P Candolfi, F Andrews, V Mather, C McGee, J Goldstein

**Affiliations:** 1 The Liverpool Heart and Chest Hospital, Liverpool, UK; 2 Edwards Lifesciences, Nyon, Switzerland

## Introduction

The aim of this study was to determine the safety and efficacy of treating patients using a novel intravenous continuous glucose monitoring (CGM) system (GlucoClear™ Edwards Lifesciences). The practical benefit of controlling blood glucose in the critically ill remains contentious, largely because of the lack of tools to adequately measure and therefore manage levels in real time. A system that is able to provide instant, constantly calibrated, accurate values is a major bonus to ICU care and we report here the first clinical use of a novel CGM system directly used to manage postoperative glycaemia.

## Methods

All consecutive consenting adult patients undergoing cardiac surgery involving cardiopulmonary bypass and requiring postoperative insulin (>98% of patients) were enrolled in the study with a target enrolment of 100. Blood glucose was measured via a dedicated peripheral intravenous catheter with values reported every 5 minutes. The primary outcome was the number of data points in the target glycaemic control range (4.4 to 8.0 mmol/l), using a dynamic insulin protocol. Secondary endpoints include mean glucose levels, time in range and number of hypoglycaemic and hyperglycaemic episodes.

## Results

For the first 45 patients, mean age was 67.7 years (male 52.8%), 54% had undergone valve surgery with or without CABG and the majority (67%) had no history of diabetes. The CGM sensor was typically sighted in the forearm or hand (90.5%) and was resited on 7.5% occasions. Median monitoring time in the ICU was 28.4 hours. Mean glycaemic value was 6.8 mmol/l. From the possible 18,609 glucose values (one per 5 minutes), 16,808 values were recorded (90.3%). Overall, 13,389/16,808 values (79.6%) were within the target range. No hypoglycaemic episodes (≤2.2 mmol/l) were recorded at any point. There were 219/16,808 values (1.3%) in the hyperglycaemic range (≥10.0 mmol/l).

## Conclusion

This new CGM system enabled significantly improved glycaemic control despite the challenges of working with an entirely new system of glucose control in a unit with over 200 nurses. Performance notably improved with experience and also highlighted that even previously validated dynamic algorithms will need to be refined to maximise the benefit of GGM. Overall, the evidence from the first applied clinical use of this novel CGM showed that proper safe, tight glycaemic control can be achieved. Further investigations are required to demonstrate the reduction in morbidity and mortality using CGM, and we plan to embark on further studies to address this.

